# Automating Colon Polyp Classification in Digital Pathology by Evaluation of a “Machine Learning as a Service” AI Model: Algorithm Development and Validation Study

**DOI:** 10.2196/67457

**Published:** 2025-07-31

**Authors:** David Beyer, Evan Delancey, Logan McLeod

**Affiliations:** 1 Department of Lab Medicine and Pathology University of Alberta Edmonton, AB Canada; 2 NGIS (Australia) Victoria Australia; 3 Deptartment of Environmental Studies University of Victoria Victoria, BC Canada

**Keywords:** AI, AI models, applicability, artificial intelligence, automated detection, cancer, cancer screening, colon cancer, colon, colon cancer screening, colon polyp, detection, develop, development, digital pathology, effectiveness, imaging, machine learning, model, pathologist, pathology, screening, whole slide imaging

## Abstract

**Background:**

Artificial intelligence (AI) models are increasingly being developed to improve the efficiency of pathological diagnoses. Rapid technological advancements are leading to more widespread availability of AI models that can be used by domain-specific experts (ie, pathologists and medical imaging professionals). This study presents an innovative AI model for the classification of colon polyps, developed using AutoML algorithms that are readily available from cloud-based machine learning platforms. Our aim was to explore if such AutoML algorithms could generate robust machine learning models that are directly applicable to the field of digital pathology.

**Objective:**

The objective of this study was to evaluate the effectiveness of AutoML algorithms in generating robust machine learning models for the classification of colon polyps and to assess their potential applicability in digital pathology.

**Methods:**

Whole-slide images from both public and institutional databases were used to develop a training set for 3 classifications of common entities found in colon polyps: hyperplastic polyps, tubular adenomas, and normal colon. The AI model was developed using an AutoML algorithm from Google’s VertexAI platform. A test subset of the data was withheld to assess model accuracy, sensitivity, and specificity.

**Results:**

The AI model displayed a high accuracy rate, identifying tubular adenoma and hyperplastic polyps with 100% success and normal colon with 97% success. Sensitivity and specificity error rates were very low.

**Conclusions:**

This study demonstrates how accessible AutoML algorithms can readily be used in digital pathology to develop diagnostic AI models using whole-slide images. Such models could be used by pathologists to improve diagnostic efficiency.

## Introduction

Many important pathological diagnoses are made by expert pathologists’ careful examination of formalin-fixed paraffin-embedded tissue slides. Advances in digital microscopy have enabled large-scale digitization of glass slides at high resolution, and the adoption of whole-slide images (WSIs) for primary sign-out of pathology is increasing [1-3]. One of the main benefits of digital pathology is improved diagnostic efficiency, which is increasingly important as the field of pathology deals with increased volumes while also struggling with the recruitment of new pathologists [4,5]. The combination of a decreased workforce coupled with an increase in the volume of cases secondary to an aging population means that pathologists need to become more efficient to meet future demand. Artificial intelligence (AI) tools applied to WSIs can be used to improve the efficiency of pathologists [6-8]. Advancements in slide scanners [9], which facilitate large-scale digitization of slides, have brought about a paradigm shift in pathology. Digitization not only enhances the efficiency of pathological examinations but also bridges the gap between conventional techniques and the ever-evolving field of AI. Ultimately, the creation of WSIs is the first step in incorporating AI into the field of pathology.

One advantage of the digitization of WSIs will be the creation of libraries of high-quality labeled training data for use with machine learning (ML) algorithms [10]. Recent developments in the fields of ML and AI, such as deep learning, for computer vision and object detection–related tasks [11-13] have led to a rapid uptake of the use of these tools in computational pathology research, where their utility has been widely recognized [14-17]. ML has traditionally required massive computational power and advanced knowledge of computer science and programming languages such as Python and R [18-20]. However, with the large-scale deployment of Machine Learning as a Service (MLaaS) platforms, these barriers to entry are minimized, allowing domain-specific experts (ie, pathologists and medical imaging professionals) to make use of advanced AI/ML tools [21]. “AutoML” algorithms and cloud-based ML platforms such as Amazon’s Sagemaker and Google’s VertexAI provide affordable, easy-to-access options that reduce overall costs by allocating centralized computer resources on demand to end users [22].

Pathologists are well-positioned with the expertise and tools required to build high-quality training datasets, which are the bedrock of effective AI models, and develop real-world uses for the production of ML models. Our project examined whether a small dataset for common colon polyp entities could be used to develop a robust and accurate ML model for diagnostic purposes using an AutoML model from Google’s Vertex AI. Colon polyps are a precursor to invasive carcinoma, and a high-volume sample is encountered in the pathology lab. As many jurisdictions use screening programs to detect and remove polyps for cancer surveillance, accurate and efficient pathology diagnosis is a key part of colon cancer screening programs [23,24]. As there are relatively few diagnostic entities for colon polyps, this area is well-suited to AI screening algorithms to assist pathologists in making a rapid diagnosis. This led to the formation of our project, aiming at evaluating an MLaaS model, trained on our own institutional data, to evaluate both the ease of model development as well as model performance when compared to pathologist interpretation.

## Methods

### Overview

Alberta Precision Laboratories has a robust digital pathology slide set, used primarily for teaching. This slide set contains images of previous cases (hematoxylin and eosin–stained histology slides) that have been scanned using an Aperio GT450. The bulk of our case images came from this dataset. In addition, to increase variability within our dataset, both for hematoxylin and eosin stain quality, as well as scanner type, we also used publicly accessible WSIs from Leeds University, University of Michigan, and the Cancer Imaging Archive [25-27]. Cases (n=494) were randomly divided into 3 allocations: training (75%), validation (10%), and test (15%, Table 1).

**Table 1:** Data allocation.

Image allocation	Number of images used, n	Percent of total
Training	1110 (370 cases)	75%
Validation	150 (50 cases)	10%
Test	222 (74 cases)	15%

In Table 1, cases and their associated images were split into 75% training, 10% validation, and 15% test. Slightly increased test cases were used (that the traditional 80/10/10 split) to better evaluate model performance, and to compensate for a relatively small training set.

We focused on 3 common entities seen in colon polyps: hyperplastic polyps, tubular adenomas, and normal colon. All cases were opened in Aperio Imagescope, and manual image/patch extraction was completed at 4× and 10× objective power (40× and 100× magnification, respectively), focusing on the most representative tumor/diagnostic areas. A total of one 4× extraction/tile and two 10× extraction/tiles were used per case, for a total of 1482 images (Table 1). Tiles were chosen by a pathologist to ensure that the most representative areas of the slide were used. The extraction was carried out using Aperio-Imagescope’s built-in image extraction tool, using the embedded International Color Consortium (ICC) profile and exported in .jpeg format. The ICC profile is a method of color normalization used by Aperio (and other digital pathology vendors) to ensure the image generated from the slide matches as closely as possible to the real-world color profile of the slide. Both the scanner and image profile used in this study are fully validated and accredited for clinical use.

Images in the dataset were rescreened by a blinded pathologist to ensure that the appropriate diagnostic label had been applied and to ensure that no images from test cases were present in either the training or validation data. A total of 3 users (2 pathologists and 1 pathology resident) were involved in the tile and review process to prevent bias in the tile selection. Instructions for tile selection were to select the most appropriate tile for the diagnosis. Only tiles with perfect consensus (3 of 3 agreed) labels were chosen to be used.

A single-label image classification model with 3 labels (“hyperplastic polyp,” “tubular adenoma,” and “normal”) was then developed using Vertex AI and an autoML algorithm. Other model parameters were set to default to demonstrate a general yet easy to use model for nontechnical experts (model details are as follows: training method: “AutoML,” Objective: “Image classification (single-label),” Data-split: “Manual,” Budget: 48 node hours, Actual: 43.04 node hours, Training time: 5 h 33 min).

**Figure 1:**
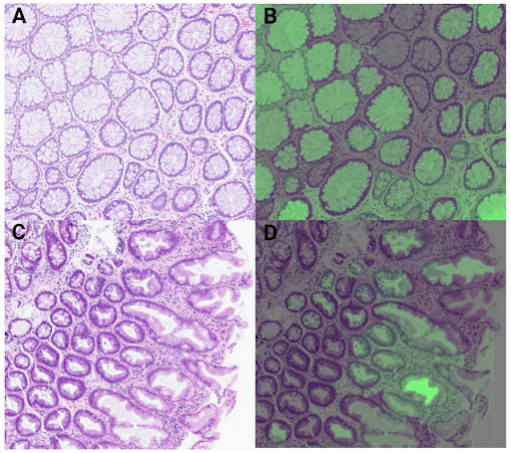
(A) Normal colon sample image. (B) Normal colon sample image with XRAI (4) overlay. (C) Hyperplastic polyp sample image. (D) hyperplastic polyp sample image with XRAI overlay. Green intensity correlates with areas of increased positive probability, that is, image segments that contribute most strongly to a given class prediction.

The AI model was tested on the “test” allocation of cases that were not part of the training or validation datasets. XRAI overlay [28] was used to view explainability and ensure that the algorithm was identifying the pertinent areas of the image (Figure 1). Model output was evaluated on a per-image basis, as opposed to a per-case basis. Results from the test data were used to evaluate precision and recall. VertexAI incorporates model evaluation and automatically generates both the precision-recall curve as well as precision-recall by threshold. An overall confidence threshold of 0.50 was chosen for the evaluation of our model.

### Ethical Considerations

Ethics approval and a waiver of consent were obtained for using deidentified case images from our institutional database. This study was approved by the Health Research Ethics Board of Alberta (HREBA.CC-23-0347).

## Results

**Figure 2:**
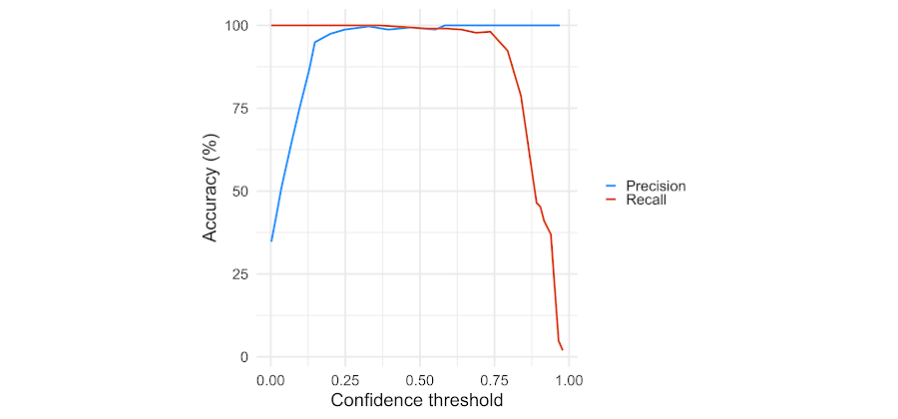
Precision-recall curve by threshold. Using a confidence value of 0.25 results in a precision of 98.7%, and recall of 100%. Using a confidence value of 0.5 results in both a precision and recall of 98.6%. Using a confidence value of 0.75 results in a precision of 100% and a recall of 97.3%. Precision sharply falls below a confidence threshold of 0.13. Recall sharply falls above a confidence threshold of 0.80. Our model output data are based on a confidence value of 0.50.

Using a CI of 0.5, the overall accuracy of the model on the test dataset was 98.4% (Table 2). Tubular adenomas and hyperplastic polyps were identified 100% of the time (66/66 and 48/48, respectively). Normal colon was identified with 97% accuracy (102/105, with 3/105 being misclassified as “hyperplastic”). Visual inspection of XRAI overlays demonstrates that the model is identifying the pertinent areas (Figure 1). Results from the classification of the test data were used to calculate both recall as well as precision, in addition to a precision-recall by threshold curve (Figure 2), and an area under the curve value of 0.99.

**Table 2:** Tubular adenoma and hyperplastic polyps were identified 100% of the time (66/66 and 48/48, respectively). Normal colon was identified with 97% accuracy (102/105).

True label ↓/Predicted label →	Tubular adenoma, n (%)	Hyperplastic polyp, n (%)	Normal colon, n (%)
Tubular adenoma	66 (100)	0 (0)	0 (0)
Hyperplastic polyp	0 (0)	48 (100)	0 (0)
Normal colon	0 (0)	3 (3)	105 (97)

## Discussion

### Principal Findings

The integration of AI in the field of digital pathology is not merely a technological advancement; it is becoming a necessity driven by the contemporary challenges that the medical community faces. Here we have demonstrated that it is relatively easy to train your own AI algorithms on your own data, as a pathologist. As ML continues to revolutionize diagnostic pathology, our study demonstrates how simple off-the-shelf AI tools can readily be used to develop effective models for improving diagnostic efficiency. The recall and precision of the developed AI model in detecting colon polyps were remarkable, approaching 100%. This exceptional performance underscores the potential of ML to transform diagnostic pathology, especially given the rapid advancements in technology, availability, and cost-effectiveness of MLaaS platforms such as Google’s VertexAI.

Previous AI applications in digital pathology, specifically in colorectal cancer screening, have shown great potential in increasing the efficiency and accuracy of diagnosis. For instance, Korbar et al [29] demonstrated that deep learning models could classify colorectal histology slides with high accuracy, bridging the gap between manual microscopic evaluations and automated assessments. Our study builds upon these foundations, offering further evidence for the efficiency and effectiveness of AI in this domain. One could say we have crafted a model that emulates the performance of an early-stage pathology resident, as the 3 entities used in this model are relatively simple; the difference is that this model took days to train, versus years for the average pathology resident. Nevertheless, there are certain limitations to consider.

First, our study had a limited sample size and did not include a complete range of diagnostic entities. With only tubular adenoma, hyperplastic polyp, and normal colon as labels, this model does not account for other critical entities like serrated adenomas, high-grade dysplasia, or carcinoma, an important histologic feature of high-risk polyps [30]; this would be a good area for future projects. AI models, particularly deep learning models, generally require large training datasets in order to generalize well in real-world scenarios [18,31]. A more extensive dataset might allow for more refined models that capture nuances and rare presentations of colon polyps, such as high-grade dysplasia. This limitation is significant, as evidenced by the emphasis on broad scope in successfully used deep neural networks for detecting colorectal cancer on WSIs [32].

Second, while our model shows promise, integrating it into real-world workflows would necessitate the inclusion of a more diverse range of clinical criteria beyond just the image data. We did not include certain clinical characteristics that would be important, like location (ie, ascending colon), which can affect diagnosis, especially for serrated lesions [23].

Third, the need to manually annotate our dataset means that we are effectively working with a “best-case scenario” data input. Real-world scenarios might present slides with artifacts, suboptimal staining, or overlapping tissues that could challenge the model’s predictions [33]. The success of AI models in clinical settings largely depends on the quality and diversity of the input data. As we only used tiles with consensus between our 3 subject matter experts (2 pathologists and 1 pathology resident), this meant a small number of imperfect tiles were excluded. These “imperfect” samples, of course, exist in the real world, and these must be interpreted as well.

Fourth, our model misclassified a number of “normal” as “hyperplastic polyp,” which appeared to be on normal colon where glands were not perfectly round. Likely inclusion of more “normal” images with different-shaped lumens would help tune the model for proper identification.

However, despite these limitations, our study exemplifies the promise of using ML as a service in histopathology. The near-perfect performance of our model in a controlled environment suggests that with adequate training data and a broader range of diagnostic entities, such platforms could soon play a significant role in augmenting the capabilities of pathologists.

Our project has shown that widely available “AutoML” algorithms, such as Google’s Vertex AI, can be applicable to the medical field, specifically in digital pathology. Our training model was robust with a sensitivity approaching 100% and a specificity of 98%. This aligns with other authors’ findings of success with AutoML algorithms in the medical field [31]. Such AI tools will help to improve the efficiency of front-line pathologists, allowing them to keep up with increasing demand and so helping to mitigate workforce constraints. For example, our model could be used to auto-generate a report, saving time for the pathologist in dictating/typing.

### Conclusion

Overall, we show that cloud-based ML platforms can produce accurate models that are specific to the field of pathology and WSIs. Furthermore, they are easy to use, with only a relatively basic level of ML knowledge required. As the medical community strives for implementation of precision medicine that should lead to improved patient outcomes [34], integration of ML tools into the realm of histopathology may soon become indispensable. Future studies with larger datasets, diverse diagnostic entities, and more real-world scenarios are warranted to further elucidate the potential of AI in this domain.

## Abbreviations

AI: artificial intelligence
ICC: International Color Consortium
ML: machine learning
MLaaS: Machine Learning as a Service
WSI: whole-slide image
